# Natural history and surgical treatment of chordoma: a retrospective cohort study

**DOI:** 10.1590/1516-3180.2014.1325628

**Published:** 2014-07-29

**Authors:** Samuel Aguiar, Wesley Pereira Andrade, Glauco Baiocchi, Gustavo Cardoso Guimarães, Isabela Werneck Cunha, Daniel Alvarez Estrada, Sergio Hideki Suzuki, Luiz Paulo Kowalski, Ademar Lopes

**Affiliations:** I MD, PhD. Surgical Oncologist, Department of Pelvic Surgery, Hospital A. C. Camargo, São Paulo, Brazil; II MD, MSc. Surgical Oncologist, Department of Pelvic Surgery, Hospital A. C. Camargo, São Paulo, Brazil; III MD, PhD. Pathologist, Department of Pathology, Hospital A. C. Camargo, São Paulo, Brazil; IV MD. Neurosurgeon, Department of Neurosurgery, Hospital A. C. Camargo, São Paulo, Brazil; V MD, PhD. Head and Neck Surgeon, Department of Head and Neck Surgery, São Paulo, Brazil; VI MD, PhD. Head of Department of Pelvic Surgery, Hospital A. C. Camargo, São Paulo, Brazil

**Keywords:** Chordoma, Surgical procedures, operative, Recurrence, Survival, Risk factors, Mortality, Cordoma, Procedimentos cirúrgicos operatórios, Recidiva, Sobrevivência, Fatores de risco, Mortalidade

## Abstract

**CONTEXT AND OBJECTIVE:**

Chordoma is a rare tumor with a high risk of locoregional recurrences. The aim of this study was analyze the long-term results from treating this pathological condition.

**DESIGN AND SETTING:**

Cohort study in a single hospital in São Paulo, Brazil.

**METHODS:**

This was a retrospective cohort study on 42 patients with chordoma who were treated at Hospital A. C. Camargo between 1980 and 2006. The hospital records were reviewed and a descriptive analysis was performed on the clinical-pathological variables. Survival curves were estimated using the Kaplan-Meier method and these were compared using the log-rank test.

**RESULTS:**

Nineteen patients were men and 23 were women. Twenty-five tumors (59.5%) were located in the sacrum, eleven (26.2%) in the skull base and six (14.3%) in the mobile spine. Surgery was performed on 28 patients (66.7%). The resection was considered to have negative margins in 14 cases and positive margins in 14 cases. The five-year overall survival (OS) was 45.4%. For surgical patients, the five-year OS was 64.3% (82.2% for negative margins and 51.9% for positive margins). In the inoperable group, OS was 37.7% at 24 months and 0% at five years.

**CONCLUSION:**

Complete resection is related to local control and definitively has a positive impact on long-term survival.

## Abstract (pt)

**CONTEXTO E OBJETIVO:**

Cordoma é um tumor raro e com alto risco de recidiva locorregional. O objetivo deste estudo foi analisar os resultados a longo prazo do tratamento dessa doença.

**TIPO DE ESTUDO E LOCAL:**

Estudo de coorte realizado em um único hospital em São Paulo, Brasil.

**MÉTODOS:**

Estudo de coorte retrospectivo com 42 pacientes com cordoma tratados de 1980 e 2006 no Hospital A. C. Camargo. Os prontuários foram revistos e foi realizada a análise descritiva das variáveis clínicas e patológicas. As curvas de sobrevida foram estimadas pelo método de Kaplan-Meier e a comparação entre elas, pelo teste de *log-rank*.

**RESULTADOS:**

Dezenove pacientes eram homens e 23, mulheres. Vinte e cinco tumores (59,5%) estavam localizados no sacro, 11 (26,2%) na base do crânio e 6 (14,3%), na coluna móvel. A cirurgia foi realizada em 28 pacientes (66,7%). A ressecção foi considerada como tendo margens negativas em 14 casos e margens comprometidas em 14 pacientes. A sobrevida global (SG) em 5 anos foi de 45,4%. Para os pacientes cirúrgicos, a SG em 5 anos foi de 64,3% (82,2% para as margens negativas e 51,9% de margens positivas). No grupo inoperável, a SG em 24 meses foi de 37,7% e 0% em 5 anos.

**CONCLUSÃO:**

A ressecção completa está relacionada com o controle local e, definitivamente, tem impacto positivo na sobrevida a longo prazo.

## INTRODUCTION

Chordoma is a rare malignant neoplasm that arises from primitive notochord remnants. It occurs exclusively in the axial skeleton and has a predilection for the sacrum (50%), base of the skull (35%) and mobile spine (15%).1-3 Although considered to be a low-grade and slow-growing tumor, a poor long-term prognosis is generally observed due to extensive local recurrences and secondary complications. Metastasis is usually reported as a late event.^4^

Proximity to neurological and other vital structures is the major challenge in making therapeutic decisions. Surgery is the mainstay of treatment and local control is better achieved with *en bloc *resection of the tumor with safe margins.^1^^,^ 2 ^,^ 4 ^,^ 5 Because of the poor responsiveness to radiotherapy, this treatment option is generally used only as adjuvant treatment after incomplete surgical resection, or in palliative management.^1^ Chemotherapy usually results in low response rates. Only a few clinical series have reported the use of chemotherapy for managing chordoma, which is generally used in the latter course of the disease and only as palliative treatment.^6^^,^ 7

Because of the rarity of this disease, only a few retrospective series have been published.

## OBJECTIVE

The purpose of this study was to describe a single institution's experience of treating chordoma.

## METHODS

This was a retrospective cohort study on all 42 consecutive patients with chordoma who were treated in the A. C. Camargo Cancer Center between 1980 and 2006. The hospital records were reviewed and a descriptive analysis was performed on the clinical-pathological variables. Survival curves were estimated using the Kaplan- Meier method and these were compared using the log-rank test.

The patients were evaluated retrospectively in relation to age, sex, duration of symptoms until diagnosis, chordoma location, symptomatology, biopsy or surgery performed before being admitted into our hospital, treatment and surgical margins, adjuvant treatment, local recurrence, systemic metastasis, complications arising from disease evolution or treatment and mortality. Patients were considered to be asymptomatic if their tumors were discovered accidentally through image examinations conducted for another reason. The surgical resections were considered to have negative margins if they had negative macroscopic or microscopic margins; and positive margins if they had positive macroscopic margins. 

### Statistical analysis

The database was set up in the Statistical Package for the Social Sciences, version 16.0 for Mac (SPSS, Inc., Chicago, IL, USA).

## RESULTS

Nineteen patients were male (45.2%) and 23 were female (54.8%). At the time of diagnosis, the median age was 47 years (range: 5-86). The median follow-up was 28.5 months and the mean was 49.3 months (range: 1-261.6). The patients' distribution according to their main characteristics is shown in Table 1. Data on the period of time from symptom onset to diagnosis were available for 37 patients (88%) with a median of 19 months (range: 1-48).

**Table 1:** Treatment data and clinical outcomes among 42 chordoma patients

n^o^	Age	Gender	Location	Margins	Adj. treatment	Recurrence	Progression	Metastasis	Death	Status
1	26	Female	Clivus	Neg	RT	No	-	No	No	NED
2	5	Male	Clivus	Neg	-	Yes	-	No	No	AWD
3	6	Female	Clivus	Neg	-	No	-	No	No	NED
4	26	Female	Clivus	Pos	-	-	Yes	No	No	DOD
5	32	Male	Clivus	Pos	-	-	Yes	No	Yes	DOD
6	37	Female	Clivus	Pos	-	-	No	No	No	AWD
7	39	Female	Clivus	Pos	RT	-	No	No	No	NED
8	50	Female	Clivus	Pos	RT	-	Yes	No	Yes	DOD
9	10	Female	Clivus	-	RT	-	Yes	No	Yes	DOD
10	18	Female	Clivus	-	CT	-	Yes	No	Yes	DOD
11	22	Female	Clivus	-	RT	-	Yes	No	Yes	DOD
12	11	Male	Cervical spine	Neg	-	No	-	No	No	NED
13	21	Female	Cervical spine	Pos	-	-	No	No	No	NED
14	52	Male	Cervical spine	Pos	RT	-	Yes	No	Yes	DOD
15	59	Female	Lumbar spine	-	CT	-	Yes	Skin	Yes	DOD
16	19	Male	Thoracic spine	Pos	-	-	Yes	No	Yes	DOD
17	28	Female	Thoracic spine	-	Palliative	-	Yes	No	No	AWD
18	56	Female	Sacrococcygeal	Neg	RT	Yes^*^	-	No	No	AWD
19	57	Male	Sacrococcygeal	Neg	-	No	-	No	No	NED
20	58	Male	Sacrococcygeal	Neg	-	Yes	-	No	Yes	DOD
21	59	Male	Sacrococcygeal	Neg	-	No	-	No	No	NED
22	63	Female	Sacrococcygeal	Neg	-	Yes	-	No	Yes	DOD
23	64	Female	Sacrococcygeal	Neg	-	No	-	No	No	NED
24	66	Female	Sacrococcygeal	Neg	RT	No	-	No	No	NED
25	68	Male	Sacrococcygeal	Neg	-	Yes	-	No	Yes	NED
26	75	Male	Sacrococcygeal	Neg	RT	Yes^*^	-	No	No	NED
27	76	Female	Sacrococcygeal	Neg	-	No	-	No	No	NED
28	28	Male	Sacrococcygeal	Pos	-	-	Yes	Lung and liver	No	AWD
29	37	Male	Sacrococcygeal	Pos	-	-	No	Lung and liver	No	AWD
30	46	Female	Sacrococcygeal	Pos	-	-	Yes	No	Yes	DOD
31	48	Male	Sacrococcygeal	Pos	-	-	Yes	Lung and liver	Yes	DOD
32	50	Female	Sacrococcygeal	Pos	RT	-	Yes	No	Yes	DOD
33	69	Male	Sacrococcygeal	Pos	RT	-	No	No	Yes	DOD
34	6	Female	Sacrococcygeal	-	Palliative	-	No	No	Yes	DOD
35	18	Female	Sacrococcygeal	-	RT	-	No	No	No	AWD
36	39	Male	Sacrococcygeal	-	RT	-	No	Bone	Yes	DOD
37	42	Male	Sacrococcygeal	-	RT	-	No	No	Yes	DOD
38	48	Female	Sacrococcygeal	-	RT	-	No	No	No	AWD
39	54	Male	Sacrococcygeal	-	RT	-	No	No	-	-
40	65	Male	Sacrococcygeal	-	RT	-	Yes	No	Yes	DOD
41	68	Male	Sacrococcygeal	-	RT	-	No	Lung	Yes	DOD
42	86	Female	Sacrococcygeal	-	RT	-	No	No	Yes	DOD

M = male

F = female

POS = positive margins

NEG = negative margins

CT = chemotherapy

RT = radiation therapy

Adj . = adjuvant

DOD = died of disease

NED = no evidence of disease

DOC = died of other causes

AWD = alive with disease

* Recurrence with new resection with positive margins.

Twenty-five tumors (59.5%) were located in the sacrum, eleven (26.2%) in the skull base and six (14.3%) in the mobile spine (three cervical, two thoracic and one lumbar). In relation to the eleven tumors of the clivus (skull base), the main symptoms were pain (six cases), diplopia or strabismus (six cases), paresthesia (two cases), dysarthria (one case) and alteration of the motor function of the tongue (one case). 

Regarding the 25 sacrococcygeal tumors, the main symptoms were pain (19 cases), presence of tumor mass in the sacrococcygeal region perceived by the patient (six cases), motor deficit or sensory neuropathy in lower limbs (six cases), constipation/bowel dysfunction (three cases) and bladder dysfunction (one case). 

Twenty-eight patients (66.7%) were considered to be in the surgical treatment group and 14 patients (33.3%) were considered to be in the nonsurgical treatment group.

### Surgical treatment

Nineteen patients (45.2%) had undergone previous surgical procedures in other institutions before admission. Only one (5.3%) had undergone complete resection previously, while 18 (94.7%) had had incomplete resections. Among the 18 patients who had previously undergone incomplete resections, 12 (66.7%) underwent salvage surgical procedures in our hospital. Five had negative margins and seven had resections with positive margins. The other six patients (33.3%) were considered to present operable disease, based on image analysis. Since these patients showed persistent disease that was measurable on images, they were considered to be in the nonsurgical group.

Twenty-eight patients (66.7%) were included in the surgical group of treatment. The surgical resection was considered to present negative margins in 14 cases (50%) and positive margins in the other 14 cases (50%). 

The tumor site influenced the extent of the resection, since neurological structures are more involved when tumors are located in the clivus or mobile spine, rather than in the sacral location. Regarding the resection of the 16 sacral tumors, ten (62.5%) were considered to have negative margins and six (37.5%) were considered to have positive margins. Among the eight skull base tumor resections, three (37.5%) were considered to have negative margins and five (62.5%) were considered to have positive margins. Out of the six patients with mobile spine tumors, only four underwent surgical treatment. Just one (25%) had negative margins (cervical spine tumor) and the other three (75%) had positive margins (two cervical and one thoracic spine tumors). When we grouped the patients with mobile spine and clivus tumors, we found that the sacral tumors had higher rates of negative margins (62.5% versus 33.3%), although this did not reach statistical significance (P = 0.25).

Adjuvant treatment was performed with radiotherapy in nine cases (32.1%), of which four had negative margins (one skull base and three sacral tumors) and five had positive margins (two skull base, one mobile spine and two sacral tumors). 

Neurological deficit was the most notable surgical morbidity. Out of the sixteen patients who underwent sacral resections, three (18.8%) had urinary incontinence, three (18.8%) had fecal retention and two (12.5%) had urinary retention. Among the eight skull base resections, one (12.5%) developed strabismus and two (25%) persistent swallowing disorders.

### Nonsurgical treatment

Fourteen patients (33.3%) did not undergo surgical treatment in our hospital. Twelve (28.6%) patients had locally advanced tumors that were considered unresectable and the last two (4.7%) received only palliative support because of their poor clinical conditions. Of these, six patients had undergone surgery in another hospital, with incomplete resection. The tumors were considered unresectable based on imaging studies, in which the tumor was seen to involve the S1 sacral level in eight cases, had extensive skull base infiltration in three cases, and involved levels from C3 to T2 in one case. Ten patients (23.8%) were administered palliative radiotherapy and two (4.8%) were administered palliative chemotherapy.

### Radiotherapy

Radiotherapy was used for 19 patients of this sample: four patients with negative margins, five patients with positive margins and ten patients with inoperable disease.

The type of radiotherapy currently used in our institution is intensity-modulated radiation therapy (IMRT). 

### Recurrence

Among the 14 patients who underwent resection with negative margins, six (42.8%) had local recurrence. Five were sacral tumors and one had received adjuvant radiotherapy. The remaining patient had a skull base tumor and did not receive adjuvant radiotherapy.

In the group with positive margins, four patients (28.5%) had stable disease during the follow-up, seven (50%) had only local progression and three developed metastases (21.4%): one with pulmonary disease alone and two with both local progression and distant disease (pulmonary and bone; pulmonary and hepatic). In the group that was considered inoperable, there were three patients (21.4%) that developed systemic metastasis (one skin and one lung). 

From analysis on the group with negative margins, the five-year progression-free survival rate was 74%. The primary tumor site also did not influence the risk of recurrence (P = 0.11). Regarding adjuvant radiotherapy, no conclusion could be reached because only four patients (25%) with negative margins received adjuvant radiotherapy. 

### Overall survival

**Figure 1:**
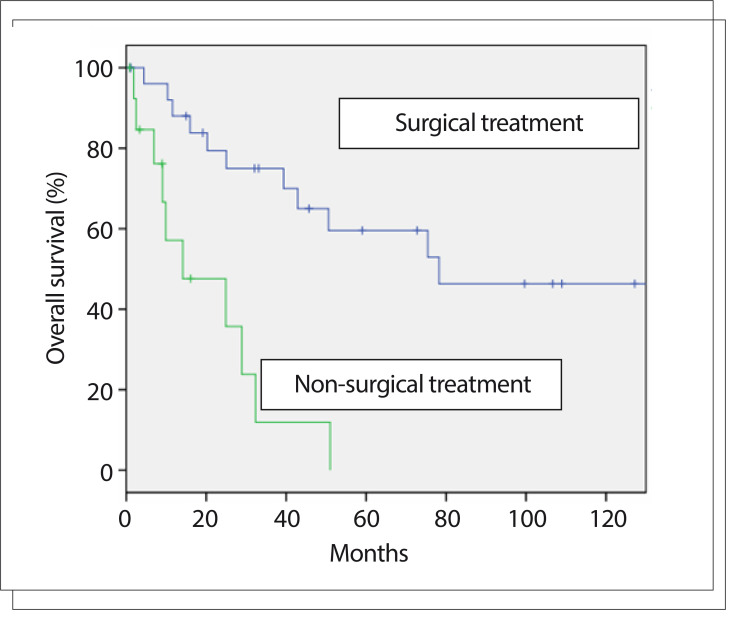
Kaplan-Meier curves for overall survival from surgical and nonsurgical treatment (P < 0.001).

**Figure 2:**
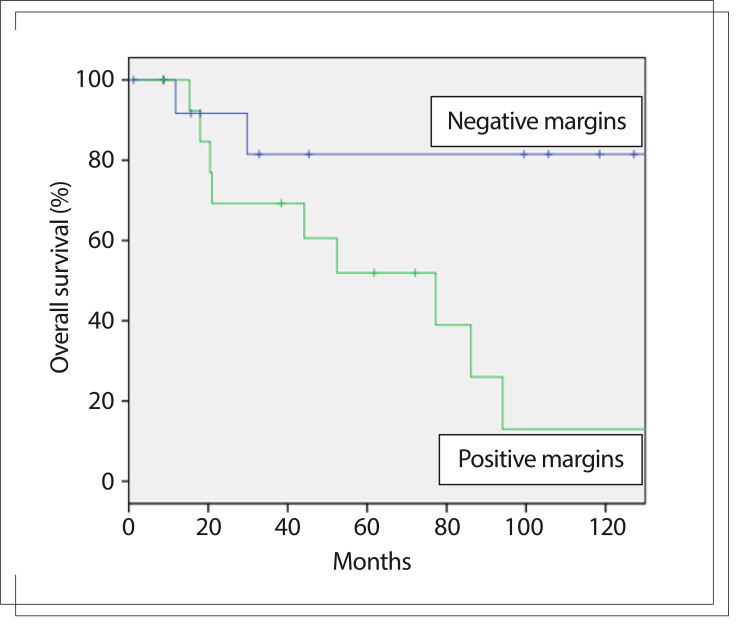
Kaplan-Meier curves for overall survival from resection with negative margins and resection with positive margins (P = 0.021).

From the analysis on the whole sample, surgical treatment (Figure 1, P < 0.001) and surgical resection with negative margins (Figure 2, P = 0.021) were the only variables that influenced the risk of death in univariate analysis (Figures 1 and 2). Other variables like sex (P = 0.92), previous tumor manipulation (P = 0.16), tumor location (P = 0.87) and adjuvant radiotherapy (P = 0.845) did not influence overall survival.

Regarding the 14 patients who received nonsurgical/palliative treatment, one patient submitted to palliative support was lost from the follow-up. The median survival in this group was 9.7 months (range: 1.0-50.3). Of these, ten had undergone radiotherapy, two received chemotherapy alone and two received only best supportive care.

The median lengths of survival of the 10 patients who underwent radiotherapy, two patients who underwent chemotherapy and patient with palliative support were respectively 9.76, 13.0 and 7.4 months. There were no difference in survival between radiotherapy and chemotherapy in the nonsurgical group (P = 0.316).

Age did not affect survival. When we divided patients into two groups based on the median age, there was no statistical difference in overall survival between patients over and under the age of 47 years.

The five-year overall survival for all patients was 45.4%. Among the patients who underwent a surgical procedure, the five-year overall survival was 64.3% (82.2% for resections with negative margins and 51.9% for resections with positive margins). In the inoperable group, survival at 24 months was 37.7% and at five years, all patients had died due to the disease.

## DISCUSSION

Chordoma is a rare malignant bone tumor originating in the axial skeleton, although it is the most common sacral primary neoplasm.8,9 The sacrum accounts for 50% of the primary sites for chordoma, followed by the skull base (35%) and mobile spine (15%).1-3 Almost the same distribution was also seen in our study (59.5% located in the sacrum, 26.2% in skull base and 14.3% in the mobile spine).

Because of the rarity of this disease, most of the previous clinical reports are based on retrospective series, with a long period of follow-up and with various types of treatment.^2^ When patients present with symptoms, they usually have radicular pain and sensory disorders relating to nerve root compression. The nonspecific symptoms often account for the diagnosis delay, with a median time period of 12 to 24 months.^1^^,^ 10 Our series corroborated these findings, with a median time period of 12 months before the diagnosis was confirmed. 

Surgery is the mainstay of treatment. Wide *en bloc *resection with adequate bone and soft tissue margins is the primary surgical goal^2^^,^ 6 ^,^ 7 ^,^ 11 and is the main prognostic factor for local recurrence and an important predictor of mortality.^12^^-^ 15 However, sometimes, wide margins are very difficult to attain because these tumors are located at sites that are difficult to access, with high rates of complications and sequelae.

Kaiser et al.^11^ reported that local failure correlated with surgical tumor margin violation. They showed a local recurrence rate of 28% for patients who had completed *en bloc* resection and 64% if the tumor capsule was violated. York et al.^7^ reported a statistically significant difference in local failure between patients who underwent radical resection (2.3 years) and those who had incomplete excision (eight months). Bergh et al.^2^ demonstrated that local control was highly improved with more aggressive surgery. Only 17% of patients with wide margin resection developed local recurrences. On the other hand, local recurrences occurred in 81% of the patients who underwent intralesional or marginal surgery. The latter authors also showed that local recurrence was significantly associated with an increased risk of metastasis and tumor-related death.

In our report, surgical resection was the only variable that had a positive impact on overall survival. Patients who underwent resections with negative margins showed a better prognosis then those with positive margins. No patient with negative margins developed distant recurrence, even after local recurrence. Surgical resection had a critical value regardless of the primary site of the tumor.

The role of radiotherapy as primary or adjuvant treatment for chordoma has been investigated.^16^^-^ 20 Some authors, such as York et al.,^7^ have reported prolonged progression-free survival with radiotherapy for patients with subtotal resection, whereas others have reported that this had little effect. Because of the small sample that underwent adjuvant radiotherapy in the present study, our report is too limited to formulate any conclusion. In our institution, IMRT is used. Some authors have described potential benefits from carbon ion radiotherapy, but we do not have this technology in our institution.^21^^,^ 22

The quality of primary treatment is reflected in the surgical margins attained in the deﬁnitive surgery and seems to be critical for the ﬁnal outcome. Among the patients who underwent resection with negative margins, 62.5% of them did not develop local recurrences. This factor was correlated with better five-year overall survival (84.4%). Among the 13 patients who had undergone incomplete resections in other institutions, seven (53.8%) had resections with negative margins in our hospital, with an obvious impact on survival for these patients. This finding supports the idea that primary surgery should be performed within a specialized multidisciplinary cancer center. 

This study has some limitations because it was conducted retrospectively, but it will certainly help other researchers and surgeons to better understand this rare disease. It also raises some questions about the definitive role of adjuvant radiotherapy after incomplete resection, and inspires investigation about the role of other adjuvant treatments such as tyrosine kinase inhibitors. These issues might need to be addressed by further studies.

## CONCLUSION

The primary goal in treating chordoma is still adequate surgical resection, and every effort needs to be made towards enabling complete removal of sacral, spinal and skull base chordomas. Complete resection is related to local control and definitively has a positive impact on survival.
